# Sequence Knowledge on *When* and *What* Supports Dual-Tasking

**DOI:** 10.5334/joc.76

**Published:** 2019-07-19

**Authors:** Fang Zhao, Robert Gaschler, Lisa Schneider, Roland Thomaschke, Eva Röttger, Hilde Haider

**Affiliations:** 1 University of Hagen, DE; 2 University of Freiburg, DE; 3 University of Cologne, DE

**Keywords:** sequence learning, stimulus-response sequence, temporal sequence, action effect of response, SRTT

## Abstract

The constraints in overlapping response selection have been established in dual-tasking studies with random sequence of stimuli and responses as well as random stimulus onset asynchrony (SOA). While this approach makes it possible to control for advance activation of upcoming stimuli or responses, it leaves open whether such preparatory processing can indeed influence dual-task performance. We investigated whether and how the sequence of stimuli and responses and the sequence of SOAs can be learned and used under dual-tasking. In each trial, participants (*N* = 28 in Experiment 1 and *N* = 30 in Experiment 2) were first presented with a random two-choice task followed by a four-choice Serial Reaction Time Task (SRTT), presented in a sequence of length four (position sequence). The SOA (timing) sequence also had length four. In test phases, one or both of the sequences were randomized. Results showed that both position and timing sequences were learned and supported dual-task performance, suggesting that predictive processing with respect to timing and identity of stimuli and responses can help to circumvent the response selection bottleneck constraints. Furthermore, in contrast to previous work on acquisition of interval sequences in single tasking, we found that the sequence of *what* (i.e. stimulus) and the sequence of *when* (i.e. interval between two tasks) contributed independently to performance.

## Introduction

Despite being a massively parallel system, the brain is capable of producing precise serial output (as in playing an instrument or typing). This “problem of serial order” (Lashley, 1951) can be approached from different perspectives. On the one hand, work on sequence learning (e.g., Schuck, Gaschler, & Frensch, 2012; Shin, 2008) and representations of serial order (e.g., Botvinick & Bylsma, 2005; Botvinick & Plaut, 2006) has dealt with representations and processes securing precision of serial output with respect to order and timing based on sequence knowledge. On the other hand, work on the response selection bottleneck (Pashler, 1994) has dealt with the constraints that avoid parallel selection of responses despite parallel activation – for instance, when multiple stimuli are presented and multiple responses are required within the same trial. In addition, early work has documented that responses of different tasks can be *executed* in temporally overlapping manner if they belong to different modalities (such as vocal and manual responses; cf. Pashler & Christian, 1994), while responding to two tasks with the same modality (e.g., two manual responses) can cause delays.

In many studies on the response selection bottleneck (e.g., Pashler, 1984; Schumacher & Schwarb, 2009), participants are first presented with the stimulus of one choice reaction task (e.g., one out of two possible stimuli) followed by the stimulus of the second choice reaction task (i.e., one out of four possible stimuli) after a variable delay. The finding that response times (RTs) in Task 1 is prolonged for shorter delays has led to different interpretations. Structural bottleneck accounts suggest that response selection of Task 2 has to wait until response selection of the Task 1 is finished (Pashler, 1994). Accordingly, studies with stimulus onset asynchrony (SOA) variation are labeled as Psychological Refractory Period (PRP) paradigm. Processing of the Task 2 stimulus or activation of the Task 2 response (Hommel, 1998) might occur in parallel to response selection in Task 1. Yet, response selection for Task 2 has to wait for Task 1 response selection to be finished. This increase of RT in Task 2 is called “PRP effect”. Alternatively to a gating mechanism allowing for only one response selection at a time, it has been proposed that the prolongation reflects that the two response selection processes can occur in parallel, but share capacity (Tombu & Jolicoeur, 2003). Cognitive-control models of dual-task performance (e.g., Hazeltine, Ruthruff, & Remington, 2006; Logan & Gordon, 2001; Meyer & Kieras, 1997; Miller, Ulrich, & Rolke, 2009; Salvucci & Taatgen, 2008; Tombu & Jolicoeur, 2003) propose that the bottleneck is strategic in nature and serves to hinder crosstalk between the two simultaneously conducted tasks. That is, this class of models assumes that the two tasks can be processed in parallel. However, serial processing in multitasking conditions occurs when it leads to higher efficiency (e.g., Miller et al., 2009).

In studies using the PRP paradigm, advance processing of Task 2 (i.e. stimulus processing or response activation) is based on the presented Task 2 stimulus. Yet, despite random order of stimuli and responses, it is not given that Task 2 processing starts with Stimulus 2 presentation. On the one hand, eyetracking work has revealed anticipatory processing (fixations to potential stimulus positions prior to stimulus onset) even with random sequences of stimulus positions (Marcus, Karatekin, & Markiewicz, 2006). On the other hand, anticipation of the time point of stimulus presentation (Shin, 2008) and response preparation (Bausenhart, Rolke, Hackley, & Ulrich, 2006) have been documented. This suggests that Task 2 processing might start prior to Stimulus 2 presentation. With random sequences in choice reaction tasks, it is difficult to assess such preparatory processes and their RT consequences: Trials in which stimulus and time point of presentation do (not) match the preparatory processes might average out in random sequences. This changes when the Serial Reaction Time Task (SRTT; Nissen & Bullemer, 1987) is combined with the PRP setup.

In the SRTT, participants react to the location of a symbol (i.e., an asterisk) on the screen by pressing the spatially corresponding key (four locations on the screen, four keys). Unbeknownst to the participants, the stimuli and responses follow a predictable sequence. Sequence knowledge leads to shorter RTs when this sequence is intact rather than when it is replaced by a sequence that the participants have not practiced. This RT benefit is obtained even when participants do not become aware of the repeating sequence (Nissen & Bullemer, 1987; Schumacher & Schwarb, 2009). Participants can acquire the sequence of stimuli (Haider, Eberhardt, Esser, & Rose, 2014) of response locations (Willingham, Wells, Farrell, & Stemwedel, 2000) and of the sequence of delays between response and next stimulus (Shin, 2008). Thus, different aspects of upcoming task processing can be prepared for in advance. Using a Stroop-like cognitive conflict paradigm, Perlman and Tzelgov (2006) found participants automatically acquire sequence knowledge in the SRTT and automatically use it to predict the upcoming stimulus and response. Furthermore, in a negative priming setup (i.e., when an irrelevant stimulus on one trial becomes relevant on a later trial), participants have been shown to acquire sequence knowledge on upcoming to-be-ignored positions, allowing for more efficient processing of the relevant stimulus position (Cock, Berry, & Buchner, 2002). This suggests that sequence learning with the SRTT can be used to shape automatic advance preparation with respect to identity of stimulus or response as well as timing.

The PRP paradigm and the SRTT have rarely been combined in one setup. Schumacher and Schwarb (2009) let participants react manually to the stimulus position with the spatially corresponding key (four keys in four positions). In the same trial, a high vs. low tone required a verbal response. Schumacher and Schwarb found that implicit learning of the fixed and repeating sequence in the SRTT was present either when the stimuli in the two tasks were presented with a delay or when they were instructed at different priority. If the stimuli were presented simultaneously at equal priority, no sequence learning was obtained. Using three different SOAs (50 ms, 125 ms, 200 ms) in Experiment 3, they obtained additive effects of sequence knowledge and SOA. In line with the PRP effect, participants were faster with longer as compared to shorter SOAs. In line with that sequence knowledge could be used to speed up performance, participants were faster in fixed sequence blocks compared to when the practiced sequence was replaced by an unpracticed order. The work by Schumacher and Schwarb suggests that by presenting the SRTT stimulus at a variable SOA after a random two-choice task stimulus can be used to study whether predictability of the sequence of stimuli and responses in the SRTT can be used for advance preparation (i.e., response activation, cf. Hommel, 1998). This advance preparation might profit from temporal predictability. While in Schumacher and Schwarb (2009) the sequence of SOAs was random, we trained participants with material that allowed to predict which stimulus would be presented as well as when it would be presented.

The knowledge about *what* to respond to (e.g., Koch, 2007) and *when* to respond (e.g., Thomaschke & Dreisbach, 2013) has been shown to facilitate the performance on reaction time tasks in single-tasking. While in many dual-tasking experiments in the lab, the sequence of stimuli and responses and the SOAs are random, this is rarely the case in everyday life, where tasks (such as playing piano) contain sequential regularities in time that are learned and used to sustain performance (Botvinick & Bylsma, 2005; Clegg, DiGirolamo, & Keele, 1998; Deroost & Soetens, 2006; Schiffer, Waszak, & Yeung, 2015). Here we examine whether and how (a) sequence knowledge about upcoming stimuli and responses and (b) sequence knowledge about the delay of stimulus onset can be used to improve performance despite potential constraints of simultaneous response selection (i.e., the response selection bottleneck). Putatively, advance information about upcoming stimuli and responses could lead to advance preparation by reducing bottlenecks (e.g., Luria & Meiran, 2003). Advance preparation might ease process-based as well as content-based dual-tasking problems (cf. Koch & Jolicoeur, 2006, 2007). If features of the stimuli or responses of the two tasks overlap, advance preparation might reduce crosstalk (cf. Koch, 2009).

Binding of stimuli and responses across tasks might disrupt retrieval of a particular response even when there is no overlap in content between the two tasks (Halvorson, Ebner, & Hazeltine, 2013; Logan & Gordon, 2001). A (predictable) temporal delay as well as a predictable stimulus sequence might ease the problem of selecting the correct response based on a stimulus activation that could match responses in either of the tasks (cf. Logan & Gordon, 2001).

Currently it is not clear to what extent participants acquire and use timing knowledge integrated with or independent of specific stimuli and responses. On the one hand, the co-occurrence can be learned. Consistent pairing of stimulus and stimulus onset leads to fast RTs (Thomaschke, Hoffmann, Haering, & Kiesel, 2016). Stadler (1995) documented that temporal disruption can impede sequence learning. Shin and Ivry (2002) found that temporal sequence learning affects performance only when the sequence of responses was left intact. This might be taken to suggest that participants can learn what to expect when and make use of this knowledge only if the pairing is left intact. On the other hand, learning about temporal contingencies can include abstract levels such as which task is associated with which delay (Aufschnaiter, Kiesel, Dreisbach, Wenke, & Thomaschke, 2018). Thus, when timing and identity of an upcoming stimulus are predictable in the PRP setup, these two sources of predictability might exert integrated and/or independent effects.

Taken together the above studies leave open whether and how sequences of stimuli and of timing can be learned and used in the PRP-setup. Although the work by Schumacher and Schwarb (2009) shows that predictable sequences of stimuli and responses can be acquired in a PRP-setup and used to speed up performance, their work used random SOAs and therefore does not show, whether predictable timing is learned and used as well. With respect to learning of timing in the SRTT, we can only draw upon the single-task work (e.g., Shin, 2008) suggesting that the timing of stimuli can be acquired and used to prepare for the specific upcoming stimulus (rather than for an upcoming stimulus in general). The current work therefore tested whether participants can acquire sequence knowledge with respect to identity and timing of upcoming stimuli and responses. It further tested whether these two aspects of predictability can support performance independently or are used in an integrated manner. Participants were trained on a sequence of fixed and repeating stimuli and responses as well as a predictable SOA sequence. In the test phase, we compared performance on the practiced sequences with performance when one or both of the repeating sequences were replaced by randomly ordered sequences. Acquisition of the sequence knowledge on identity and on timing should lead to faster performance, when the practiced sequence is left intact rather than replaced by a random sequence. As sequences of the identity and timing were of the same length, participants could in principle acquire an integrated sequence of what to expect when. If this was the case, switching one aspect (either identity or timing) to random in the test phase should deteriorate performance to a similar extent as replacing just one sequence by random order.

## Overview of the Experiments

**Figure 1:**
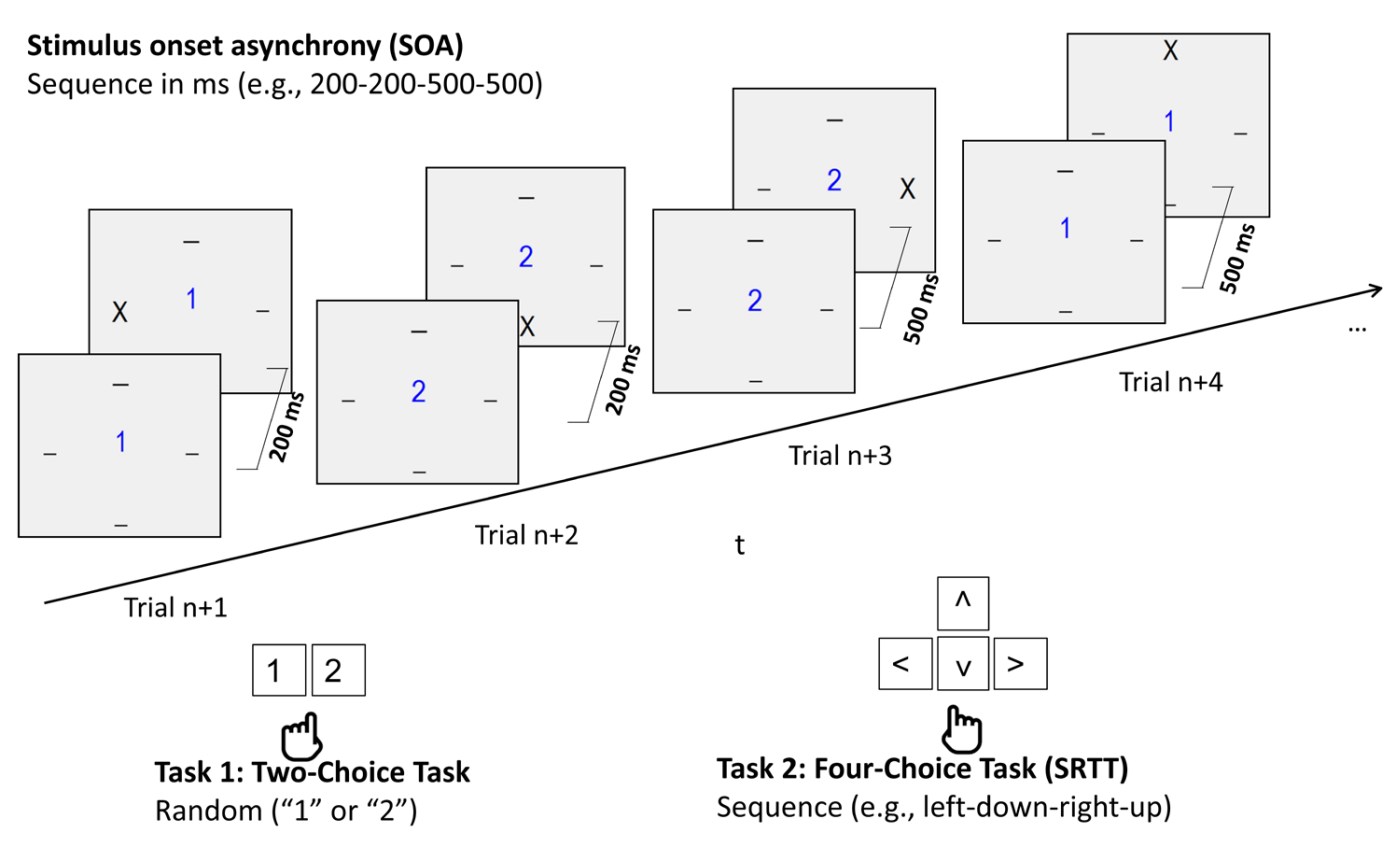
The dual-tasking setup. On each trial, participants completed a two-choice task (Task 1; stimuli and responses: 1 or 2) and a four-choice task (Task 2, SRTT; stimuli X at the upper, lower, left or right position; response with corresponding arrow key). The order of stimuli in the two-choice task was random. The delay between stimulus onset in the two-choice task as well as the sequence of positions in the four-choice task followed fixed sequences of length four.

Building on Schumacher and Schwarb (Experiment 3, 2009), we used the SRTT paradigm to examine whether preparatory processing can improve performance despite potential constraints of simultaneous response selection (i.e., the response selection bottleneck)^1^. Accordingly, we investigated whether the sequence of stimuli and responses as well as the sequence of SOA can be learned and used in dual-tasking. While Shin (2008) studied learning of temporal sequences in a single task SRTT, we trained participants with a predictable delay between stimulus onset of a (randomly sequenced) two-choice task and the SRTT. We paired a visual-manual two-choice task with random stimulus sequence with the visual-manual SRTT. In both experiments, participants first practiced a fixed repeating sequence of stimulus- and response positions and a sequence of delays between the two visual-manual tasks (see Figure 1), before being tested on either type of sequence knowledge. This was done by transferring participants to blocks in which either or both types of sequences were randomized (i.e. random sequence of timing or random sequence of stimuli) to observe whether randomization is detrimental to performance.

## Experiment 1

### Method

#### Participants

Twenty-eight participants (18 female) participated in Experiment 1 (*M*_age_ = 30.3 years, *SD* = 15.7). The mean age of participants was higher than in many laboratory studies in cognitive psychology as students at FernUniversität in Hagen (state-run distance teaching university in Germany) are older and more heterogeneous in age than students at other universities. All participants had normal or corrected-to-normal vision acuity. Participants gave their written informed consent before their participation. The experiment was part of a Bachelor of Science thesis and participates were offered course credit in compensation.

#### Stimuli, task and apparatus

Each trial contained a two-choice task (Task 1) and a four-choice task (Task 2, SRTT), separated by a SOA. For the two-choice task, participants were supposed to press the number key *1* or *2* with the index and middle finger of the left hand, in response to the numbers *1* or *2* presented centrally in blue on a light grey background on a 17.3 inch laptop. If participants pressed the wrong key, they received visual error feedback, displayed at the position of the stimulus. The sequence of stimuli in the two-choice task was random. After the two-choice task stimulus was displayed, the four-choice task followed after a variable SOA of either 200 ms or 500 ms. In most of the blocks, the SOA followed a sequence of length four, which was either alternated on each trial, or alternated every second trial. The two timing sequences were 200 ms – 200 ms – 500 ms – 500 ms or 200 ms – 500 ms – 200 ms – 500 ms. One of the two versions was randomly picked for each participant.

The SRTT was the four-choice task. For each participant, a sequence of length four (each position occurring once) was randomly picked. In most of the blocks the SRTT stimulus and response position followed this repeating sequence. The target was the letter *X*, which was displayed in black in one of four positions (left, right, up, down) around the screen center. It had to be responded to by pressing either the *left, right, up* and *down* arrow on the key pad with the right hand.^2^ If participants pressed the wrong key, an error indicator would appear at all four positions. For each participant, a fixed four element sequence of the four spatial positions was generated at random (e.g., left – down – right – up in Figure 1). There was no response deadline. Participants could only go to the next trial if they finished both tasks. It is important to note that all RTs were calculated from the onset of the particular stimulus of interest. Thus, the clock for measuring SRTT RT started after the SOA (i.e. when the SRTT stimulus was presented).

#### Procedure

The experiment was implemented in Lazarus (Lazarus Team, 1993–2016). At the beginning, participants were informed that the four-choice task would sometimes come with a fixed repeating sequence of stimulus positions, and that the delay between the two tasks could also come in a fixed sequence. We instructed participants to respond to each of the two stimuli as quickly as possible. We made explicit that participants should not wait for the second stimulus in order to respond to both stimuli together. Rather we asked them to respond to each of the two stimuli independently and as quickly as possible. Different from in Schumacher and Schwarb (Experiment 3, 2009), we used two visual-manual tasks. Furthermore, we neither instructed participants that responding to the second stimulus first would be considered as an error nor did we provide error feedback for such reversal trials. As our sequence of stimulus positions was much shorter (length 4 instead of length 12), we assumed that stronger sequence knowledge might develop and lead to strong advance preparation. Therefore, securing that SRTT responses cannot occur prior to two-choice task responses might have constituted third task demanding substantial executive control. If we had demanded that participants would always respond to the two-choice task first, RTs in the SRTT might have reflected a mixture of the amount of sequence-knowledge based advance-preparation (i.e., response activation, cf. Hommel, 1998) and efforts to schedule the usage of this activation in response execution. We therefore opted for not implementing this constraint.

Participants worked through six blocks of 144 trials each (see Table 1). In each trial, participants received both tasks, the two-choice task and the four-choice task. The first three blocks were for practicing the position- and the timing sequence. Blocks 4 to 6 were testing blocks: There was one block in which the SOA was randomized, one block in which the sequence of positions in the four-choice task was randomized, and one block with random timing and random positions. The order of these three testing blocks (*random SOA sequence stimuli, random SOA random stimuli*, and *sequence SOA random stimuli*; see Table 1) was counterbalanced across participants. Thus, a confounding of testing and training effects was avoided. At the end of the experiment, participants were asked to report the position sequence.^3^

**Table 1:** Overview of the 3 [P]ractice blocks and 3 [T]est blocks in Experiment 1: The order of Blocks 4 to 6 was counterbalanced across participants.^3^ Seq is Sequenced and Ran is Random.

Block	1P	2P	3P	4T	5T	6T

Timing	Seq	Seq	Seq	Seq	Ran	Ran
Stimulus	Seq	Seq	Seq	Ran	Seq	Ran

### Results

Participants had *M* = 3.4% (*SD* = 1.8%) errors for the two-choice task and *M* = 2.2% (*SD* = 2.1%) for the four-choice task. For the post experimental sequence report, eleven participants (39.3%) reported the practiced sequence of length 4 correctly (while tolerating different starting points in the experiment). We excluded the first four trials of each block and the trials with errors, as well as trials in which both RTs exceeded 2 sec. Performance in the practice blocks (Blocks 1 to 3; all with fixed stimulus sequence and fixed SOA sequence) is reported in the Appendix (see Table A1). In the Appendix, we also report analyses of response order. A variant of the main analyses while excluding trials in which the SRTT response was registered prior to the two-choice task response is reported in the Appendix as well.

Before reporting on the impact of sequence knowledge on SRTT performance (our main result), we provide analyses on the impact of SOA on RTs in either task and report on potential effects of disruption of the SRTT sequence on the (randomly sequenced) two-choice task.

#### Test of the impact of SOA

Ahead of our main analyses, we examined whether the SOA manipulation led to RT differences in the test phase and whether these were in line with the PRP effect (i.e., Pashler, 1994). As the SRTT was presented second, the PRP effect would predict an impact of SOA on RTs in the SRTT (shorter RTs for longer SOA). The two-choice task should not be influenced by SOA. In contrast to the PRP effect, RTs of the two-choice task (Task 1) at SOA 200 ms (*M* = 664 ms, *SD* = 177 ms) were significantly shorter than at SOA 500 ms (*M* = 815 ms, *SD* = 142 ms), *t*(27) = –9.36, *p* < .001, *d* = 0.94. Consistently with the PRP effect, RTs for the SRTT (Task 2) at SOA 500 ms (*M* = 596 ms, *SD* = 154 ms) were significantly shorter than at SOA 200 ms (*M* = 703 ms, *SD* = 198 ms), *t*(27) = 8.54, *p* < .001, *d* = 0.60.

#### RTs of the two-choice task (Task 1)

Given that RT of the first task was influenced by the SOA, it was relevant to check whether disruption of the position sequence or the timing sequence of the SRTT influenced the two-choice task. A two-factorial ANOVA with block type and SOA (200 ms vs. 500 ms) was performed to examine whether sequences of the stimulus and the timing in the SRTT also affected the two-choice task. Only a main effect of SOA was revealed, *F*(1, 27) = 87.88, *p* < .001, *η_p_^2^* = .77, suggesting that RTs of the two-choice task were shorter in 200 ms SOA condition (*M* = 664 ms, *SD* = 184 ms) than in 500 ms SOA condition (*M* = 817 ms, *SD* = 157 ms). It might suggest SOA influenced which task was responded to first. We found neither a main effect of block type, *F* < 1, nor an interaction effect, block type × SOA, *F*(1.65, 44.44) = 3.31, *p* = .06, *η_p_^2^* = .11 (here and elsewhere we applied Greenhouse Geisser-correction when appropriate). Thus, disrupting the stimulus sequence or the timing sequence in the SRTT did not influence the behaviour on the random two-choice task.

#### RTs of the SRTT (Task 2)

**Figure 2:**
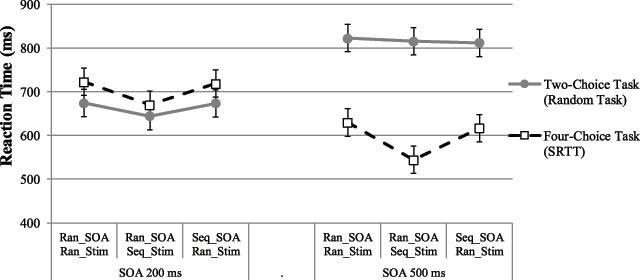
Mean reaction times (RTs) for the two-choice task and the four-choice task (SRTT) in three types of blocks in Experiment 1 plotted with 95% within subjects confidence intervals based on the error variance of block (Masson & Loftus, 2003). The RTs are calculated from the onset of the particular stimulus of interest. The exact values can be found in Appendix Table A2.

The two-factorial repeated measure ANOVA with block type and SOA (200 ms vs. 500 ms) was performed to examine learning of the stimulus-response sequence (see Figure 2 and more details in Table A2). The main effect of block type, *F*(2, 54) = 12.48, *p* < .001, *η_p_^2^* = .32, suggested shorter RTs in the condition involving a repeating stimulus sequence compared to other conditions. Contrast analyses showed that the RTs in the *random SOA sequence stimuli* test block (*M* = 607 ms, *SD* = 185 ms) were shorter than in the *random SOA random stimuli* test block (*M* = 676 ms, *SD* = 182 ms, *F*(1, 27) = 21.24, *p* < .001, *η_p_^2^* = .44) and the *sequence SOA random stimuli* test block (*M* = 667 ms, *SD* = 176 ms, *F*(1, 27) = 16.58, *p* < .001, *η_p_^2^* = .38). Our data revealed no significant difference between the *random SOA random stimuli* test block and the *sequence SOA random stimuli* test block (*p* = .63). Thus, sequence knowledge about the position of stimuli and responses enhanced performance. However, with the design of Experiment 1, we could not detect an effect of timing sequence knowledge.

The main effect of SOA, *F*(1, 27) = 72.09, *p* < .001, *η_p_^2^* = .73, suggested the responses were shorter with long SOA than short SOA. The interaction of block type × SOA, *F*(1.50, 40.44) = .63, *p* = .03, *η_p_^2^* = .12, indicated that the RT differences between short and long SOA was especially large when there was a repeating stimulus sequence (see Figure 2, more details in Table A2). Presumably, stimulus sequence knowledge could be accessed and/or used better at long SOAs, as there was more time for accessing knowledge about the upcoming stimulus.

### Discussion

Providing participants with a two-choice task with random stimulus sequence followed by a predictable stimulus in the SRTT at a predictable interval, Experiment 1 targeted whether sequence knowledge could lead to advance preparation in dual-tasking. Our results suggest that participants could acquire sequence knowledge about stimulus and response positions in the SRTT and use this sequence knowledge for speeding up performance. Sequence knowledge can be acquired in the SRTT despite a task with random stimulus sequence was additionally present. This is in line with earlier work detailing when and why adding a random task to the SRTT disrupts sequence learning. Schumacher and Schwarb (2009) reported that sequence learning was present when the stimuli in the two tasks were presented with a delay and/or the two tasks were instructed at different priorities. They attributed lack of sequence learning in case of simultaneous presentation of stimuli at equal priority to detrimental effect of the response selection bottleneck on sequence learning. In line with Schmidtke and Heuer (1997), Röttger et al. (2019) developed an alternative account, attributing the detrimental effects of dual-tasking on sequence learning to effects of the randomness being added to the SRTT. If participants fail to process stimuli and responses in the two tasks in strict separation, adding a task with random stimulus sequence makes predicting the upcoming (compound) stimuli more difficult. Yet, in the current experiment, we provided temporal separation between the stimuli of the two tasks which should have reduced the impact of added randomness.

Experiment 1 could not provide evidence for temporal sequence learning, as we had to rely on comparing performance in the two test blocks with random stimulus sequence (one with predictable timing and the other with random timing sequence). Given that Stadler (1995) documented that temporal disruption can impede sequence learning and that Shin and Ivry (2002) found that temporal sequence learning affects performance only when the sequence of responses was left intact, the current design was only a weak test of temporal sequence learning in dual-tasking. A fully crossed design was therefore used in Experiment 2.

## Experiment 2

Experiment 1 left open whether predictable timing of the presentation of the SRTT stimulus can be learned and used to support performance in dual-tasking. We hypothesized that participants could learn and use the position sequence as well as the timing sequence. RT in the SRTT should be shorter with the practiced as compared to a random position sequence. Likewise, predictable SOAs should lead to shorter RTs as compared to blocks with random SOAs. To test these hypotheses, we included a test phase that allowed to target timing sequence knowledge and positions sequence knowledge in all combinations. The test phase in Experiment 2 was based on a full 2 (fixed timing sequence vs. random timing sequence) × 2 (fixed sequence of stimuli and responses in the four-choice task vs. random sequence) design, using an additional block that mimicked the practice phase (i.e. with sequential stimuli and SOAs). The order of the blocks was counterbalanced to avoid the training effect (see Table 2).

**Table 2:** Overview of the 3 [P]ractice blocks and 4 [T]est blocks in Experiment 2: The order of Blocks 4 to 7 was counterbalanced across participants. Seq is short for Sequence and Ran is Random. Block 7 is the extra block in Experiment 2.

Block	1P	2P	3P	4T	5T	6T	7T

Timing	Seq	Seq	Seq	Seq	Ran	Ran	Seq
Stimulus	Seq	Seq	Seq	Ran	Seq	Ran	Seq

### Method

#### Participants

Thirty-one new participants (19 female) took part in Experiment 2 (*M*_age_ = 35.6 years, *SD* = 12.6) and were rewarded with 8€. Data were reported on only 30 participants due to technical problems that occurred during data collection of one participant.

#### Task and procedure

In contrast to Experiment 1, we added an extra test block with a fixed sequence of stimuli and a fixed sequence of SOAs (see Table 2). The order of test blocks was counterbalanced across participants.

### Results

The average error rate for the two-choice task was 1.7% (*SD* = 1.2%), and for the four-choice task it was 2.6% (*SD* = 1.7%). 35.5% of the participants reported the position sequence correctly. The timing sequence was reported correctly by 25.8% of the participants. We used the same exclusion criteria as in Experiment 1 and focused on the data from the 4 test blocks.

#### Test of the impact of SOA

In contrast to the PRP effect, RTs of two-choice task (Task 1) at SOA 200 ms (*M* = 698 ms, *SD* = 181 ms) were significantly faster than at SOA 500 ms (*M* = 853 ms, *SD* = 138 ms), *t*(29) = –10.24, *p* < .001, *d* = 0.96. In agreement with the PRP effect, RTs of SRTT (Task 2) at SOA 500 ms (*M* = 612 ms, *SD* = 193 ms) were significantly shorter than at SOA 200 ms (*M* = 740 ms, *SD* = 233 ms), *t*(29) = 12.02, *p* < .001, *d* = 0.60. Consistent with Experiment 1, there was a strong effect of SOA in Task 1 and in Task 2.

#### RTs of the two-choice task (Task 1)

In the two-choice task, we obtained a main effect of timing sequence, *F*(1, 29) = 7.82, *p* = .009, *η_p_^2^* = .21. Potentially, the predictable SRTT stimulus at predictable interval served as a predictable action effect of the two-choice task (see Discussion). As in Experiment 1, the main effect of SOA, *F*(1, 29) = 104.84, *p* < .001, *η_p_^2^* = .78, suggested the RTs of the two-choice task were shorter in 200 ms SOA condition than in 500 ms SOA condition. There was no main effect of stimulus sequence, *F*(1, 29) = 1.37, *p* = .25, *η_p_^2^* = .05. This was not surprising, as the two-choice task came with a random sequence in all blocks. As in Experiment 1, there was no interaction: Stimulus sequence × timing sequence, *F* < 1, stimulus sequence × SOA, *F*(1, 29) = 2.82, *p* = .10, *η_p_^2^* = .09, timing sequence × SOA, *F*(1, 29) = 1.55, *p* = .22, *η_p_^2^* = .05, and stimulus sequence × timing sequence × SOA, *F* < 1.

#### RTs of the SRTT (Task 2)

**Figure 3:**
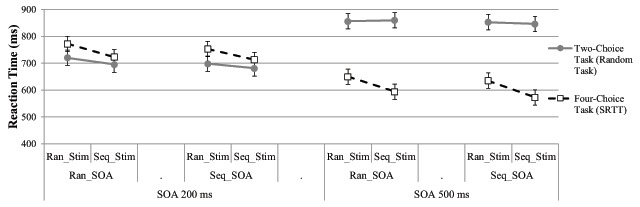
Mean RTs with timing sequence, stimulus sequence and SOA factors for the two-choice task and the four-choice task (SRTT) in Experiment 2 plotted with 95% within subjects confidence intervals based on the error variance of timing sequence × stimulus sequence (Masson & Loftus, 2003). The exact values can be found in Appendix Table A4.

In Experiment 2, we could test the impact of stimulus sequence (fixed sequence of stimuli and responses in the four-choice task vs. random sequence), the impact of timing sequence (fixed timing sequence vs. random timing sequence) and SOA (200 ms vs. 500 ms) in a 2 × 2 × 2 design (see Figure 3). The ANOVA showed a main effect of stimulus sequence, *F*(1, 29) = 34.40, *p* < .001, *η_p_^2^* = .54, suggesting that RTs in conditions that involved sequence stimulus conditions were shorter than in conditions that involved random stimulus. The main effect of timing sequence, *F*(1, 29) = 8.68, *p* = .006, *η_p_^2^* = .23, indicated RTs in conditions involving a fixed timing sequence were shorter than in conditions involving random timing. The main effect of SOA, *F*(1, 29) = 136.30, *p* < .001, *η_p_^2^* = .83 suggested the responses were shorter with long SOA than short SOA. There was no interaction: Stimulus sequence × timing sequence, *F* < 1, stimulus sequence × SOA, *F*(1, 29) = 1.48 *p* = .23, *η_p_^2^* = .05, timing sequence × SOA, *F* < 1, stimulus sequence × timing sequence × SOA, *F* < 1. The results suggest that both the stimulus and timing sequence knowledge could be used independently of one another.

In the Appendix (see Figure A3) we reported a quintile analysis checking for potential trade-offs between processing of the SRTT and the two-choice task. The fastest two-choice trials had the shortest SRTT RTs. Furthermore, effects of sequence knowledge were present in the SRTT RTs of the trials with the fastest two-choice task responses.

### Discussion

Experiment 2 tested whether and how participants could acquire and use a fixed timing sequence and position sequence in the SRTT paired with a two-choice task with random stimulus sequence. Results suggested that participants can acquire and use the position sequence (replication of Experiment 1) and additionally the timing sequence under dual-tasking. While Shin and colleagues (Shin, 2008; Shin & Ivry, 2002) reported that people learn sequences of response stimulus intervals under single task conditions, we could show that participants can use a predictable delay between the onset of the stimulus in a task with a random stimulus sequence and the onset of the SRTT stimulus. Importantly, according to our results, the timing sequence was used to support performance even when the stimulus sequence in the SRTT was switched to random in the test phase – and so did the stimulus sequence when the timing sequence was switched to random. Thus, in contrast to prior work on implicit sequence learning with longer sequences in single-task setups (Shin & Ivry, 2002), we showed that timing sequence and sequence of stimuli and positions could be used independently from each other. This finding seems difficult to reconcile with accounts attributing adaptation to the properties of the task material in encoding of episodic traces by joining timing and other task events (e.g., stimuli and responses; cf. Schmidt, 2013; Schmidt & Weissman, 2016). Yet, it fits to reports that participants can (in addition to information about which specific stimulus is to be expected when) acquire temporal information on more abstract levels such as which task is to be expected when (cf., Aufschnaiter et al., 2018).

Interestingly, a fixed repeating sequence of SOA between stimulus onsets in the two tasks did not only speed up reactions in the SRTT (Task 2), but also in the two-choice task (Task 1, which always contained a random stimulus sequence) in *short* SOA (see Figure 3). One potential explanation for this backward effect of sequence knowledge on the two-choice task comes from the literature on action-effect anticipation (for reviews, see Hommel, 2013). More specifically, the onset of the four-choice task stimulus was a temporally predictable action effect of the response to the two-choice task in sequenced SOA blocks. Predictable action effects, in turn, can speed up response selection. Likewise, responses are associated with their temporal interval (Dignath, Pfister, Eder, Kiesel, & Kunde, 2014). Consistent pairing of stimulus and stimulus onset leads to faster RTs (Thomaschke et al., 2016).

## General Discussion

Most prior dual-task studies have used random sequences of stimuli in either task and (if present) random delays between stimulus presentations in either task. Yet, in many everyday contexts the timing and order of stimuli in different tasks are not random. Combining the PRP setup with the SRTT, we targeted whether participants could acquire and use sequence knowledge about the identity and timing of the upcoming stimulus. We reasoned that despite potential constraints on parallel response selection (e.g., Pashler, 1994, Schumacher & Schwarb, 2009), sequence knowledge might lead to advance activation of upcoming stimuli and responses (cf. Hommel, 1998).

Both experiments showed that a fixed repeating sequence of stimulus- and response positions can speed up dual-task performance. Experiment 2 showed that a timing sequence was acquired and used independent of the stimulus sequence. By this, we extend and further specified earlier findings on sequence learning in single-task setups to dual-tasking. Shin and Ivry (2002) as well as Shin (2008) suggested that timing sequences are closely connected to stimulus- or response sequences. While a position sequence could be learned with or without a predictable timing sequence, the presence of predictable time patterns facilitated the expression of the acquired sequence knowledge. Our results suggest that in dual-tasking participants can independently use knowledge about the timing and identity of upcoming stimuli. Timing knowledge could be used to speed responding even when the stimulus sequence was switched to random. Predictability of timing and identity of stimuli had additive effects.

The debate on mechanisms behind the detrimental effect of dual-tasking on sequence learning can deliver tentative explanations for this lack of integration of timing- and identity-sequence knowledge. Different authors have suggested that sequence learning is disrupted in dual-tasking, because participants fail to separate the processing of the SRTT and the additional task (cf. Rah, Reber, & Hsiao, 2000; Röttger et al., 2019; Schmidtke & Heuer, 1997, 1997). Therefore, combining the SRTT with a task with random sequence of stimuli compromises learning and predicting upcoming (compound) stimuli. As delays in stimulus presentation may serve as one means to separate the processing of the two tasks (cf. Schumacher & Schwarb, 2009), our setup might have provided means and motivation for such a separation. There was neither a predictive relationship between identity of the two-choice task stimulus and the upcoming SRTT stimulus nor between the identity of the two-choice task stimulus and the timing of the upcoming SRTT stimulus. Thus, the timing sequence might have been learned and represented with respect to a predictable order in the delay between Task 1 and Task 2 rather than with respect to delays being followed by specific SRTT stimuli. As the above work on detrimental effects of dual-tasking on sequence learning showed that sequence learning was spared when there was a predictive relationship between the stimuli in the SRTT and the other task, future work should test whether this would lead to the acquisition of an integrated timing sequence.

In addition to providing evidence for sequence learning of timing in dual-tasking, our work extends the knowledge with respect to usage of sequence knowledge in PRP setups. Using three different SOAs (50 ms, 125 ms, 200 ms), Schumacher and Schwarb (2009, Experiment 3) obtained additive effects of sequence knowledge and SOA. Our results matched these findings though we used a larger span of SOA, a larger sample, and both tasks in our setup were visual-manual. In either study, participants were faster with longer as compared to shorter SOAs and in fixed sequence blocks compared to when the practiced sequence was replaced by an unpracticed order. Notably, despite that the effect of sequence knowledge on RT was numerically larger with longer SOAs, no significant interaction was obtained by Schumacher and Schwarb (2009). On the one hand, this is surprising given that longer SOAs should grant more time for stimulus processing and response activation, so sequence knowledge should have stronger effects on trials with longer SOAs. A similar interpretation (more time = more advance activation) has been put forward to explain larger effects of sequence knowledge on RT with an response stimulus interval of 250 ms rather than a zero in a single-tasking setup (Destrebecqz & Cleeremans, 2001). On the other hand, the small N of the Schumacher and Schwarb Experiment 3 (*N* = 13) and the modest variation of SOA might be taken to suggest that further tests are necessary on the question of whether longer SOA leads to stronger sequence knowledge effects. In the current study we increased N, the range of SOA manipulated and used a shorter sequence, which should have led to stronger sequence knowledge and more being granted for using it to prepare for the SRTT stimulus and response. Nevertheless, we did not find a robust impact of SOA on the usage of sequence knowledge either. Conceivably, our shorter SOA provided sufficient time for activating the upcoming SRTT stimulus and response so that more time would not yield additional benefits. However, this perspective does not fit well with the finding that the longer SOA yielded a larger proportion of trials in which the SRTT was responded to first. Potentially, participants used the longer SOA to retrieve the upcoming SRTT event while this was less efficient with a short temporal separation between the randomly sequenced two-choice task and the SRTT.

Constraints on parallel response selection can be taken to suggest that RT in the task presented first should remain unaffected by SOA manipulations while the reaction time of the second task is affected (cf. Pashler, 1994). Similar to previous reports (e.g., Jentzsch, Leuthold, & Ulrich, 2007; Logan & Gordon, 2001; Miller et al., 2009; Schumacher & Schwarb, 2009, Experiment 3), we observed that RT2 was shortened by longer SOA while RT1 was prolonged. In both experiments, we observed that RT1 was substantially longer with long SOA as compared to short SOA. Conceivably, predictability in timing and identity led participants to prepare for upcoming stimuli and responses in advance and use the presented stimulus as a trigger for actual responding (cf. Hommel, 1998). With the prolonged RT1 in the long SOA condition, participants could likely have perceived both stimuli before responding (rather than initiating the response to Task 1 before the Task 2 stimulus was perceived). Potentially, pairing two visual-manual tasks might have led participants to prepare upfront, but respond once both stimuli are presented. While modality overlap might have eased learning of the predictable SOA, it might have made it difficult to respond to the tasks independently (as instructed). Future studies comparing the overlapping vs. non-overlapping pairings (like in Schumacher & Schwarb, 2009, as well as Röttger et al. 2019) can help to test this.

Furthermore, different experimental setups have provided evidence for that Task 2 processing can influence Task 1 processing, suggesting that a potential response selection bottleneck (e.g., Pashler, 1994) does not hinder activation processes (cf. Hommel, 1998). For instance, Durst and Janczyk (2019) have detailed how Task 2 providing a NoGo-trial or a trial spatially (in)compatible to Task 1, can influence Task 1 processing in a backward manner. While the response selection bottleneck model (Pashler, 1994) can be taken to suggest that RT1 should not be influenced by SOA, we found shorter RT1 when the delay of the Task 2 stimulus was predictable rather than random. Furthermore, RT1 was shorter with shorter delay as compared to longer delay. In order to further secure the interpretations of such results, future studies should vary whether response order in the two tasks is free or fixed. In summary, our study suggests that sequence knowledge on when and what can support dual-tasking. Timing sequence and position sequence can be applied independently.

## Data Accessibility Statement

The software, the original materials, the raw data, and the aggregated data are available at www.osf.io/8g6j5.

## Additional File

The additional file for this article can be found as follows:

10.5334/joc.76.s1 Appendix A. Full results and additional analyses (i.e., response order, excluding trials in which the SRTT response was provided first, and quintile analysis).

## Footnotes

The raw data and the experiments are in osf.io/8g6j5. The software of the experiment and the original materials are available to other researchers for purposes of replicating the procedure or reproducing the results.

Measurement from mass-produced keyboards can be delayed due to hardware timing features, device driver issues, and interface delays (Plant & Turner, 2009). In general, the time delay of the keyboard is additive. Therefore regular keyboards can be sufficient to compare RT means between different conditions (see also Hilbig, 2016; Ulrich & Giray, 1989).

As the design that only Blocks 4 to 6 was counterbalanced across participants, block 3 *SOA sequence stimulus sequence* thus cannot be used as the testing block due to the training effect.
