# Supplementary material for: Sequence Knowledge on When and What Supports Dual-Tasking
Source: J Cogn. 2019 Jul 19;2(1):18. doi: 10.5334/joc.76 (PMC6640251; doi:10.5334/joc.76)
Supplement: Appendix A. — Full results and additional analyses (i.e., response order, excluding trials in which the SRTT response was provided first, and quintile analysis). [file joc-2-1-76-s1.pdf]

Appendix

Table A1

Mean reaction times (RTs) and standard deviation of the mean in practice blocks in Experiment 1.

Block	1Practice		2Practice		3Practice	
	M (ms)	*SD* (ms)	*M* (ms)	*SD* (ms)	*M* (ms)	*SD* (ms)
**Two-choice task**						
RTs	760	128	761	192	751	186
**Four-choice task (SRTT)**						
RTs	723	259	684	245	640	228

Table A2

Mean reaction times (RTs) and standard deviation of the mean in three testing blocks in Experiment 1.

Block	Ran SOA Ran Stim		Ran SOA Seq Stim		Seq SOA Ran Stim	
	M (ms)	*SD* (ms)	*M* (ms)	*SD* (ms)	*M* (ms)	*SD* (ms)
**Two-choice task**						
SOA 200 ms	674	200	644	165	673	190
SOA 500 ms	823	178	816	130	812	165
Average	749	184	729	184	741	170
**Four-choice task (SRTT)**						
SOA 200 ms	723	208	670	195	719	209
SOA 500 ms	630	159	544	180	617	148
Average	676	182	607	185	668	176

Table A3

Mean reaction times (RTs) and standard deviation of the mean in practice blocks in Experiment 2.

Block	1Practice		2Practice		3Practice	
	M	*SD*	*M*	*SD*	*M*	*SD*

	(ms)	(ms)	(ms)	(ms)	(ms)	(ms)
			**Two-choice task**			
RTs	815	149	807	174	789	162
			**Four-choice task (SRTT)**			
RTs	780	229	733	229	697	234

Table A4

Mean reaction times (RTs) and standard deviation of the mean in four testing blocks with SOA 200 ms and SOA 500 ms in Experiment 2.

Block	Ran_SOA Ran_Stim		Ran_SOA Seq_Stim		Seq_SOA Ran_Stim		Seq_SOA Seq_Stim	
	M (ms)	*SD* (ms)	*M* (ms)	*SD* (ms)	*M* (ms)	*SD* (ms)	*M* (ms)	*SD* (ms)
				**Two-choice task**				
SOA 200 ms	720	198	695	177	698	199	681	170
SOA 500 ms	856	160	860	133	852	145	846	140
Average	790	173	777	152	774	168	762	147
				**Four-choice task (SRTT)**				
SOA 200 ms	772	248	723	233	753	226	713	238
SOA 500 ms	650	204	594	199	635	168	573	216
Average	710	225	657	212	694	195	644	225

Analysis of response order

Response order in Experiment 1. Table A5 displays the effect of SOA on order of responding in the two tasks. The proportion of responding to SRTT first was subjected to a two-factorial ANOVA with block type (*random SOA random stimulus* vs. *random SOA sequence stimulus* vs. *sequence SOA random stimulus*) and SOA (200 ms vs. 500 ms) as factors. Participants were more likely to respond to SRTT first at long SOA (M = 5.6%, SD = 9.8%) than at short SOA (M = 3%, SD = 10%), demonstrating a main effect of SOA, $F(1, 27) = 9.68$, $p = .004$, $\eta_p^2 = .26$. There was no main effect of block type, $F < 1$. but an interaction of block type × SOA, $F(2, 54) = 7.97$, $p = .001$, $\eta_p^2 = .23$, suggested that participants responded to the four-choice task first more frequently when there was a fixed repeating position sequence in long SOA than in short SOA (8.5% vs. 2.5%).

Table A5

Proportion of trials in which the response to the SRTT (Task 2) was given prior to the two-choice task (Task 1) response in the testing blocks of Experiment 1.

	Ran SOA Ran Stim		Ran SOA Seq Stim		Seq SOA Ran Stim	
	M	*SD*	*M*	*SD*	*M*	*SD*
SOA 200 ms	2.4%	7.5%	2.5%	7.3%	4.2%	1.4%
SOA 500 ms	2.9%	3.6%	8.5%	8.4%	5.3%	1.4%
Average	2.7%	5.8%	5.5%	8.4%	4.7%	1.4%

Response order in Experiment 2. The proportion of trials in which the SRTT response was given first (see Table A6) was submitted to a three-factorial ANOVA with SOA (200 ms vs. 500 ms), timing sequence (*random vs. sequence*), and stimulus sequence (*random vs. sequence*). There was a main effect of SOA, $F(1, 29) = 16.81$, $p < .001$, $\eta_p^2 = .37$, due to a higher proportion of SOA 500 ms trials ($M = 9.9\%$, $SD = 18.6\%$) in which the SRTT response came first compared to SOA 200 ms ($M = 5.7\%$, $SD = 18.3\%$). There was no main effect of stimulus sequence, $F(1, 29) = 2.01$, $p = .17$, $\eta_p^2 = .07$, but a replicated interaction effect of stimulus sequence × SOA, $F(1, 29) = 6.12$, $p = .02$, $\eta_p^2 = .17$, suggested that effects of sequence stimulus blocks were stronger in long SOA than in short SOA (11.7% vs. 8.1%). No other effects were significant: timing sequence, $F < 1$, timing sequence × stimulus sequence, $F < 1$, timing sequence × SOA, $F(1, 29) = 3.02$, $p = .09$, $\eta_p^2 = .09$, timing sequence × stimulus sequence × SOA, $F(1, 29) = 3.02$, $p = .09$, $\eta_p^2 = .09$.

Table A6

Proportion of trials in which the response to the four-choice task (the SRTT) was given prior to the two-choice task response for the testing blocks of Experiment 2.

	Ran SOA Ran Stim		Ran SOA Seq Stim		Seq SOA Ran Stim		Seq SOA Seq Stim	
	M	*SD*	*M*	*SD*	*M*	*SD*	*M*	*SD*
SOA 200 ms	6.3%	18.1%	6.7%	20.8%	5.4%	19.1%	4.6%	15.9%
SOA 500 ms	8.0%	16.8%	11.6%	21.4%	8.2%	19.1%	11.7%	17.5%
Average	7.1%	17.3%	9.2%	21.1%	6.8%	19.0%	8.1%	17.0%

Repeating analyses while excluding trials in which the SRTT response was provided first

Results were in line with the analyses not excluding reversal trials.

Experiment 1: Test of the impact of SOA. In contrast to the PRP effect, RTs for the two-choice task (presented first in the experiment) at SOA 200 ms ($M = 659$ ms, $SD = 174$ ms) were significantly shorter than at SOA 500 ms ($M = 793$ ms, $SD = 145$ ms), $t(27) = -8.51$, $p < .001$, $d_z = 1.61$. Consistently with the PRP effect, RTs for the SRTT (presented second) at SOA 500 ms ($M = 605$ ms, $SD = 150$ ms) were significantly shorter than at SOA 200 ms ($M = 704$ ms, $SD = 198$ ms), $t(27) = 7.31$, $p < .001$, $d_z = 1.38$.

Experiment 2: Test of the impact of SOA. In contrast to the PRP effect, RTs for two-choice task (presented first in the experiment) at SOA 200 ms (*M* = 696 ms, *SD* = 178 ms) were significantly lower than at SOA 500 ms (*M* = 827 ms, *SD* = 151 ms), $t(29) = -10.67$, $p < .001$, $d_z = 1.92$. In agreement with the PRP effect, RTs for SRT-responses (presented second) at SOA 500 ms (*M* = 621 ms, *SD* = 186 ms) were significantly shorter than at SOA 200 ms (*M* = 741 ms, *SD* = 230 ms), $t(29) = 9.03$, $p < .001$, $d_z = 1.63$.Participants seemed to group their responses: they strategically postponed Task 1 at long SOA to retrieve the stimulus sequence of Task 2, which in turn led to faster Task 2 processing.

Experiment 1: RTs of the two-choice task. A two-factorial ANOVA with block type and SOA (200 ms vs. 500 ms) was performed to examine whether stimulus sequence and timing sequence affected the random task (presented first). There was a main effect of block type, $F(1.32, 35.63) = 38.89$, $p < .001$, $\eta_p^2 = .59$. Further contrast analyses indicated quicker responses in the *random SOA sequence stimuli* test block (*M* = 707 ms, *SD* = 147 ms) than in the *random SOA random stimuli* test block (*M* = 739 ms, *SD* = 185 ms), $F(1, 27) = 45.20$, $p < .001$, $\eta_p^2 = .63$, and in *sequence SOA random stimuli* test block (*M* = 732 ms, *SD* = 162 ms), $F(1, 27) = 17.32$, $p < .001$, $\eta_p^2 = .39$. No difference was revealed between *random SOA random stimuli* test block and *sequence SOA random stimuli* test block ($p = .75$). The main effect of SOA, $F(1, 27) = 26.12$, $p < .001$, $\eta_p^2 = .49$, suggested that RTs of the two-choice task were shorter in the 200 ms SOA condition (*M* = 659 ms, *SD* = 182 ms) than in the 500 ms SOA condition (*M* = 793 ms, *SD* = 158 ms). It might suggest SOA influenced which task was responded to first. We found also an interaction effect, block type × SOA, $F(2, 54) = 12.21$, $p < .001$, $\eta_p^2 = .31$. It indicated that RT differences of two-choice task between short and long SOA was larger when there was the stimulus sequence in the SRTT. The RT in Task 1 also benefitted from the sequence knowledge in Task 2.

Experiment 2: RTs of the two-choice task. In the two-choice task, we obtained a main effect of timing sequence, $F(1, 29) = 6.32$, $p = .018$, $\eta_p^2 = .18$. The main effect of SOA, $F(1, 29) = 76.85$, $p < .001$, $\eta_p^2 = .73$, suggested the RTs of the two-choice task were shorter in 200 ms SOA condition (*M* = 696 ms, *SD* = 192 ms) than in 500 ms SOA condition (*M* = 827 ms, *SD* = 171 ms). There was no main effect of stimulus sequence, $F(1, 29) = 3.60$, $p = .068$, $\eta_p^2 = .11$. Again, there was no interaction: Stimulus sequence × timing sequence, stimulus sequence × SOA, timing sequence × SOA, and stimulus sequence × timing sequence × SOA, *Fs* < 1. It indicated that disrupting the stimulus sequence, the timing sequence and SOA did not influence the behaviour on the two-choice task.

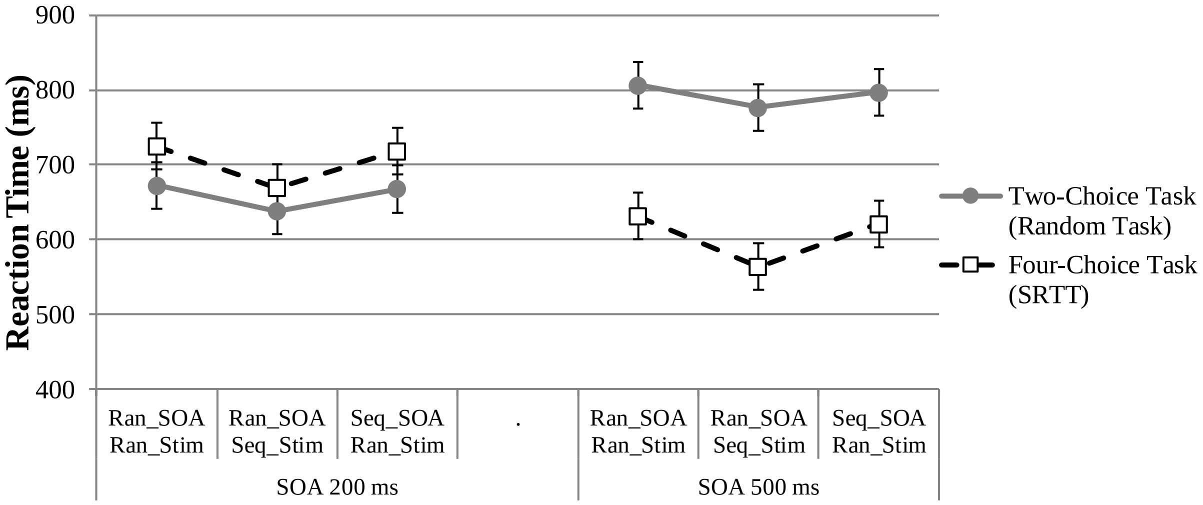

Figure A1. Experiment 1 excluding SRTT prior to two-choice task plotted with 95% within subjects confidence intervals based on the error variance of block type (Masson & Loftus, 2003).

Experiment 1: RTs of the SRTT. A two-factorial repeated measure ANOVA with block type and SOA (200 ms vs. 500 ms) was performed to examine learning of the stimulus-response sequence. The main effect of block type, $F(1.45, 39.15) = 37.58$, $p < .001$, $\eta_p^2 = .58$, suggested shorter RTs in the condition involving a repeating stimulus sequence than other conditions. Contrast analyses showed that the RTs in the *random SOA sequence stimuli* test block (M = 616 ms, SD = 180 ms) were shorter than in the *random SOA random stimuli* test block (M = 678 ms, SD = 183 ms, $F(1, 27) = 5.28$, $p = .03$, $\eta_p^2 = .16$, and the *sequence SOA random stimuli* test block (M = 670 ms, SD = 174 ms, $F(1, 27) = 50.15$, $p < .001$, $\eta_p^2 = .65$. Thus, stimulus-response sequence knowledge enhanced performance.

The main effect of SOA, $F(1, 27) = 15.35$, $p = .001$, $\eta_p^2 = .36$, suggested the responses were shorter with long SOA (M = 605 ms, SD = 160 ms) than short SOA (M = 704 ms, SD = 204 ms). The interaction of block type × SOA, $F(2, 54) = 12.95$, $p < .001$, $\eta_p^2 = .32$, indicated that the RT differences between short and long SOA was especially large when there was a repeating stimulus sequence. Presumably, stimulus sequence knowledge could be accessed and/or used better at long SOA.

Experiment 2: RTs of the SRTT. The impact of stimulus sequence (fixed sequence of stimuli and responses in the four-choice task vs. random sequence), timing sequence (fixed timing sequence vs. random timing sequence), and SOA (200 ms vs. 500 ms) was tested in a 2 × 2 × 2 design. The ANOVA showed a main effect of stimulus sequence, $F(1, 29) = 28.64$, $p < .001$, $\eta_p^2 = .50$, suggesting that RTs in conditions that involved sequence stimulus conditions were shorter than in conditions that involved random stimulus. The main effect of timing sequence, $F(1, 29) = 5.44$, $p = .027$, $\eta_p^2 = .16$, indicated RTs in conditions involving a fixed timing sequence were shorter than in conditions involving random timing. The replicated main effect of SOA, $F(1, 29) = 105.68$, $p < .001$, $\eta_p^2 = .79$ suggested the responses were shorter with long SOA (M = 622 ms, SD = 199 ms) than short SOA (M = 742 ms, SD = 242 ms). There was no interaction: Stimulus sequence × timing sequence, stimulus sequence × SOA, timing sequence × SOA, and stimulus sequence × timing sequence × SOA, $Fs < 1$. The results suggest that both the stimulus and timing sequence knowledge could be used independently of one another.

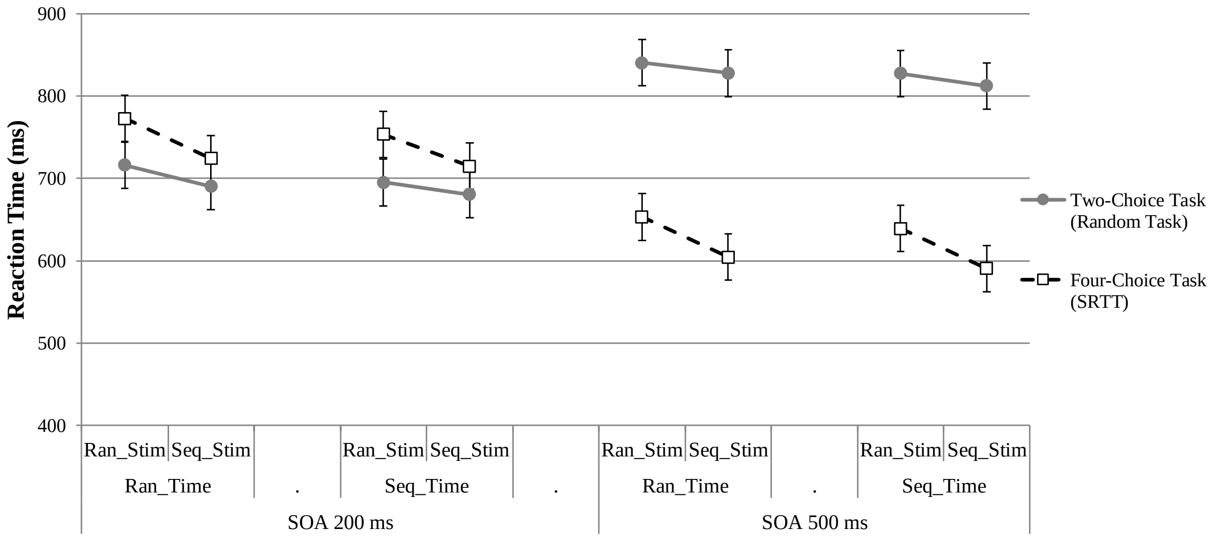

Figure A2. Experiment 2 excluding SRTT prior to two-choice task plotted with 95% within subjects confidence intervals based on the error variance of timing sequence × stimulus sequence (Masson & Loftus, 2003).

Quintile analysis for Experiment 2

The fastest two-choice trials had the shortest SRTT RTs. Furthermore, effects of sequence knowledge were present in the SRTT RTs of the trials with the fastest two-choice task responses.

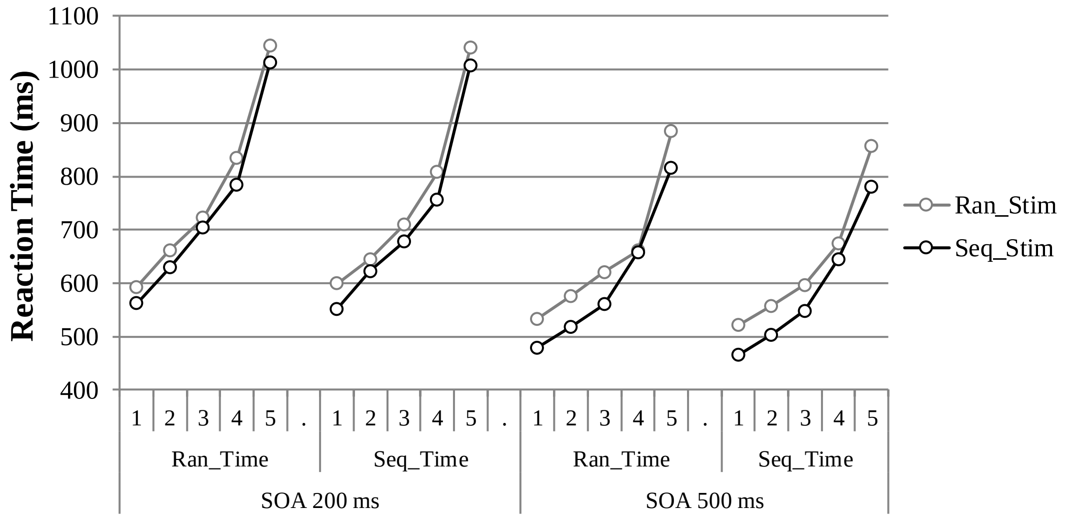

Figure A3. Quintile analysis of test phase data of Experiment 2. Quintiles were computed based on the two-choice task RT per participant, SOA, timing sequence and position sequence.
